# *SRPX2* promotes cancer cell proliferation and migration of papillary thyroid cancer

**DOI:** 10.1007/s10238-023-01113-1

**Published:** 2023-06-12

**Authors:** Haiwei Guo, Ruiqi Liu, Jiajun Wu, Shuang Li, Weiping Yao, Jiajie Xu, Chuanming Zheng, Yanwei Lu, Haibo Zhang

**Affiliations:** 1 Otolaryngology and Head and Neck Center, Cancer Center, Department of Head and Neck Surgery, Zhejiang Provincial People’s Hospital, Affiliated People’s Hospital, Hangzhou Medical College, Hangzhou, Zhejiang China; 2 Cancer Center, Department of Radiation Oncology, Zhejiang Provincial People’s Hospital, Affiliated People’s Hospital, Hangzhou Medical College, Hangzhou, Zhejiang China; 3 Graduate Department, Bengbu Medical College, Bengbu, Anhui China; 4 Key Laboratory of Endocrine Gland Diseases of Zhejiang Province, Hangzhou, Zhejiang China; 5 Clinical Research Center for Cancer of Zhejiang Province, Hangzhou, Zhejiang China

**Keywords:** *SRPX2*, Papillary thyroid cancer, Migration, Proliferation, *PI3K/AKT*

## Abstract

Thyroid cancer is the endocrine tumor with the highest incidence at present. It originates from the thyroid follicular epithelium or follicular paraepithelial cells. There is an increasing incidence of thyroid cancer all over the world. We found that *SRPX2* expression level was higher in papillary thyroid tumors than in normal thyroid tissues, and *SRPX2* expression was closely related to tumor grade and clinical prognosis. Previous reports showed that *SRPX2* could function by activating *PI3K/AKT* signaling pathway. In addition, in vitro experiments showed that *SRPX2* promoted the proliferation and migration of papillary thyroid cancer (PTC). In conclusion, *SRPX2* could promote the malignant development of PTC. This may be a potential treatment target for PTC.

**Supplementary Information:**

The online version contains supplementary material available at 10.1007/s10238-023-01113-1.

## Introduction

Thyroid cancer is one of the most common cancers, and it ranks seventh among cancers diagnosed in women [1, 2]. Its incidence has significantly increased over the past few decades. According to the difference in tumor origin and differentiation, thyroid cancer can be further divided into PTC, follicular thyroid carcinoma (FTC), medullary thyroid carcinoma (MTC), poorly differentiated thyroid cancer (PDTC), and anaplastic thyroid cancer (ATC) [3]. PTC is the most common type of thyroid cancer, accounting for about 80% of all thyroid malignancies [4]. Thyroid cancer therapy includes surgery, chemotherapy, radiotherapy, and targeted therapy. Although most thyroid cancers are curable, advanced thyroid cancers show increased incidence and mortality rates [5]. The development of systematic treatments for advanced thyroid cancer using molecular landscapes is an emerging field [6]. Fortunately, it is reported that the genomics of thyroid cancer has promoted new therapeutic targets.

*SRPX2*, also known as *sushi repeat-containing protein X-linked 2*, is located on Xq22.1. *SRPX2* was first identified as the downstream target gene of *E2A-HLA* in leukemia in 1999 [7]. The *SRPX2* protein is related to nerve development and cell growth and widely expressed in normal tissues [8, 9]. Besides, *SRPX2* is highly expressed in cancer tissues and cell lines, including glioma, mesothelioma, osteosarcoma, esophageal, gastric, lung, and colorectal cancers [8, 10–15]. Recent studies have revealed that a variety of signaling pathways are involved in the upregulation of *SRPX2* in human diseases, such as the binding of transcription factors [16, 17]. In addition, inflammation can also promote the expression levels of *SRPX2* [18]. Moreover, *SRPX2* is regulated by its upstream molecules including TGFβR1/SMAD3, NFATc3/c-JUN, MAN1 (LEM) domain containing 1 (LEMD1), miR-149, and FOXP2 [17, 19–22]. The effects of these mechanisms are not only limited to the upregulation of *SRPX2* but also regulate the biological behaviors of tumor cell proliferation, migration, and invasion. Therefore, the upregulation of *SRPX2* may be an important promoting factor for tumor development and metastasis.

*SRPX2* promotes cell migration in gastric cancer, but not cellular growth. *SRPX2* can increase the interaction between endothelial cells and tumors, regulating tumor progression and metastasis [8]. *SRPX2* overexpression plays a malignant role in colorectal cancer by regulating cell proliferation, adhesion, migration, and invasion [11]. Moreover, *SRPX2* regulates glycolytic metabolism in colon cancer cells through the *PI3K–Akt* pathway [11]. *SRPX2* also increases osteosarcoma cell proliferation by activating the Hippo signaling pathway [23]. The role of *SRPX2* in thyroid cancer, however, is unclear.

We searched for thyroid cancer biomarkers and found that *SRPX2* was upregulated in PTC. In vitro experiments also show that *SRPX2* plays a vital role in the proliferation and migration of PTC. Our study aimed to clarify the biological functions and regulatory mechanisms of *SRPX2* in PTC.

## Materials and methods

### Cell cultures

TPC1 (RET/PTC rearranged, PTC), BCPAP (BRAFV600E mutated, PTC), IHH4 (BRAFV600E mutated, PTC), and NTHY3 (Nthy-ori 3–1, normal thyroid epithelial cell) were obtained from ATCC. All cell lines were cultured in RPMI-1640 medium (HyClone, China) containing 10% fetal bovine serum (Gibco, USA). The cell incubator condition was a 5% CO_2_ atmosphere with a constant temperature of 37 °C.

### Tissue samples and clinical data collection

The Zhejiang Provincial People’s Hospital provided six pairs of formalin-fixed, paraffin-embedded thyroid cancer specimens and adjacent non-tumor specimens. We provide the basic information of the patient in Supplementary Table 1. No samples were collected from patients undergoing chemotherapy or radiotherapy. Ethical approval was obtained from the Institutional Ethical Review Board of Zhejiang Provincial People’s Hospital (Institutional Review Board number QT2022435) before commencing sample analysis.

### RNA interference

The cells were plated one day before transfection. Small interfering RNA (siRNA) transfection was performed using the RNAi transfection reagent (Invitrogen). The sequences of siRNA were as follows:

- siControl:5’-TTCTCCGAACGTGTCACGT-3’, 3’-TGCACTGTGCAAGCCTCTT-5’.
- si*SRPX2*-1:5’-GGUGAAAGAUUCUGCUGCUGAUTT-3’, 3’-AUCAGCAGAAUCUUUCACCTT-5’.
- si*SRPX2*-2:5’-CCGAGGAAAUCUUCACAUUTT-3’, 3’-AAUGUGAAGAUUUCCUCGGTT-5’.

### RNA sequencing

Trizol (Invitrogen) was used to isolate RNA from three biologically repeated siControl and three biologically repeated si*SRPX2*-1-transfected TPC1 cells after 36 h. Transcriptome expression profiling was analyzed by RNA sequencing using NovaSeq 6000 platform (Illumina) by Shanghai Bioegene Co., Ltd. After the final transcriptome was generated, StringTie (http://ccb.jhu.edu/software/stringtie/,version:stringtie-2.1.6) and ballgown (http://www.bioconductor.org/packages/release/bioc/html/ballgown.html) were used to estimate the expression levels of all transcripts and perform expression abundance for mRNAs by calculating FPKM (fragment per kilobase of transcript per million mapped reads) value. Genes differential expression analysis was performed by DESeq2 software between two different groups (and by edgeR between two samples). The genes with the false discovery rate (FDR) parameter below 0.05 and absolute fold change > 2 were considered differentially expressed genes. Differentially expressed genes were then subjected to enrichment analysis of GO functions and KEGG pathways.

### RNA preparation and real-time quantitative PCR

Twenty-four hours after transfection, total RNA was isolated by RNA-Quick Purification Kit (ES Science, China) and quantified by absorbance at OD 260 nm. Total RNA was reverse-transcribed into cDNA by using the PrimeScript RT Reagent Kit (Takara Biotechnology, China). All the PCRs were carried out using qPCR SYBR Green Master Mix (Yeasen, China). The LightCycler 480 (Roche Diagnostics) was used for the real-time PCR assays. In order to normalize the expression of mRNA, GAPDH was used as a reference. The primers used for the related genes are listed in Table 1.

**Table 1:** The primers of the genes

Gene symbol	Forward/reverse primer
GAPDH	Forward: 5'-GTCATCCATGACAACTTTGG-3' Reverse: 5'-GAGCTTGACAAAGTGGTCGT-3'
*SRPX2*	Forward: 5'-CCACATGCTACTCACCGAAGG-3' Reverse: 5'-GTAGTGCGTGGCATCTCATCT-3'
GLS2	Forward: 5'- TCTCTTCCGAAAGTGTGTGAGC-3' Reverse: 5'- CCGTGAACTCCTCAAAATCAGG-3'
S100A14	Forward: 5'- GAGACGCTGACCCCTTCTG-3' Reverse: 5'- CTTGGCCGCTTCTCCAATCA-3'
PREX2	Forward: 5'- AAGACCGAGCGGGACTATGT-3' Reverse: 5'- TGTTGAGCATTAGGTTCGGGG-3'
GREM1	Forward: 5'- CGGAGCGCAAATACCTGAAG-3' Reverse: 5'- GGTTGATGATGGTGCGACTGT-3'
LAMA4	Forward: 5'- ATGAGCTGCAAGGAAAACTATCC-3' Reverse: 5'- CTGTTTCGTTGGCTTCACTGA-3'
PTGDS	Forward: 5'- AGCACCTACTCCGTGTCAGT-3' Reverse: 5'-TGGGTTCGGCTGTAGAGGG-3'

### Western blot analysis

Western blot analysis was performed as previously described [24]. Forty-eight hours after transfection, the cells were lysed in RIPA buffer with 1% PMSF on ice, and the cell lysates were quantified using the BCA reagent (Thermo Scientific, China). The proteins were separated on 8–12% SDS–PAGE gels and electrotransferred to PVDF membranes. Here are the antibodies used for probing the membranes: GAPDH (ab8245; 1:5000; Abcam), P-PI3K (4228; 1:3000; CST), PI3K (4249; 1:3000; CST), FN1 (26836; 1:3000; CST), Epithelial–Mesenchymal Transition (EMT) Antibody Sampler Kit (9782T; CST) including E-cadherin (3195; 1:3000; CST), N-cadherin (13116; 1:3000; CST), and vimentin (5741; 1:3000; CST).

### Proliferation assays

Cells transfected with the specified siRNAs were inoculated into six-well plates (1 × 10^4^ cells each well). The cells were digested with trypsin/EDTA in suspension every other day to determine the number of cells.

### Wound healing assay

For wound healing experiments, TPC1 and IHH4 cells were incubated in six-well plates until the cell density reached 90%. In the center of the hole, a straight line was drawn with a sterile pipette tip. The wound was subsequently washed with PBS, and the cells were cultured in serum-free 1640 for 12 h. The wound area was captured by a microscope and measured by ImageJ software.

### Transwell Assay

Transwell assays were performed in Transwell plates (LABSELECT, China, 6.5 mm). Equal amounts of TPC1 and IHH4 cells were inoculated into the upper chamber containing 200 µL serum-free 1640 (5 × 10^4^ cells). The lower chamber contained 700 µL of 1640 containing 10% FBS for induction of cell migration. The cells were cultured for 24 h. The migrated cells were photographed and counted under a microscope and then analyzed by ImageJ software.

### Statistical analysis

The means and standard deviation are calculated from at least three independent experiments performed in duplicate. For statistical significance, we used GraphPad Prism 8.0 software to perform t-tests (and nonparametric tests) and one-way ANOVA (and nonparametric or mixed). Statistical methods are selected based on the sample size and sample distribution characteristics. The Kaplan–Meier method was used for the survival analysis. The significance of a statistical test was defined as *P < 0.05, **P < 0.01, ***P < 0.001.

## Results

### *SRPX2* expression levels are upregulated in PTC

**Fig. 1:**
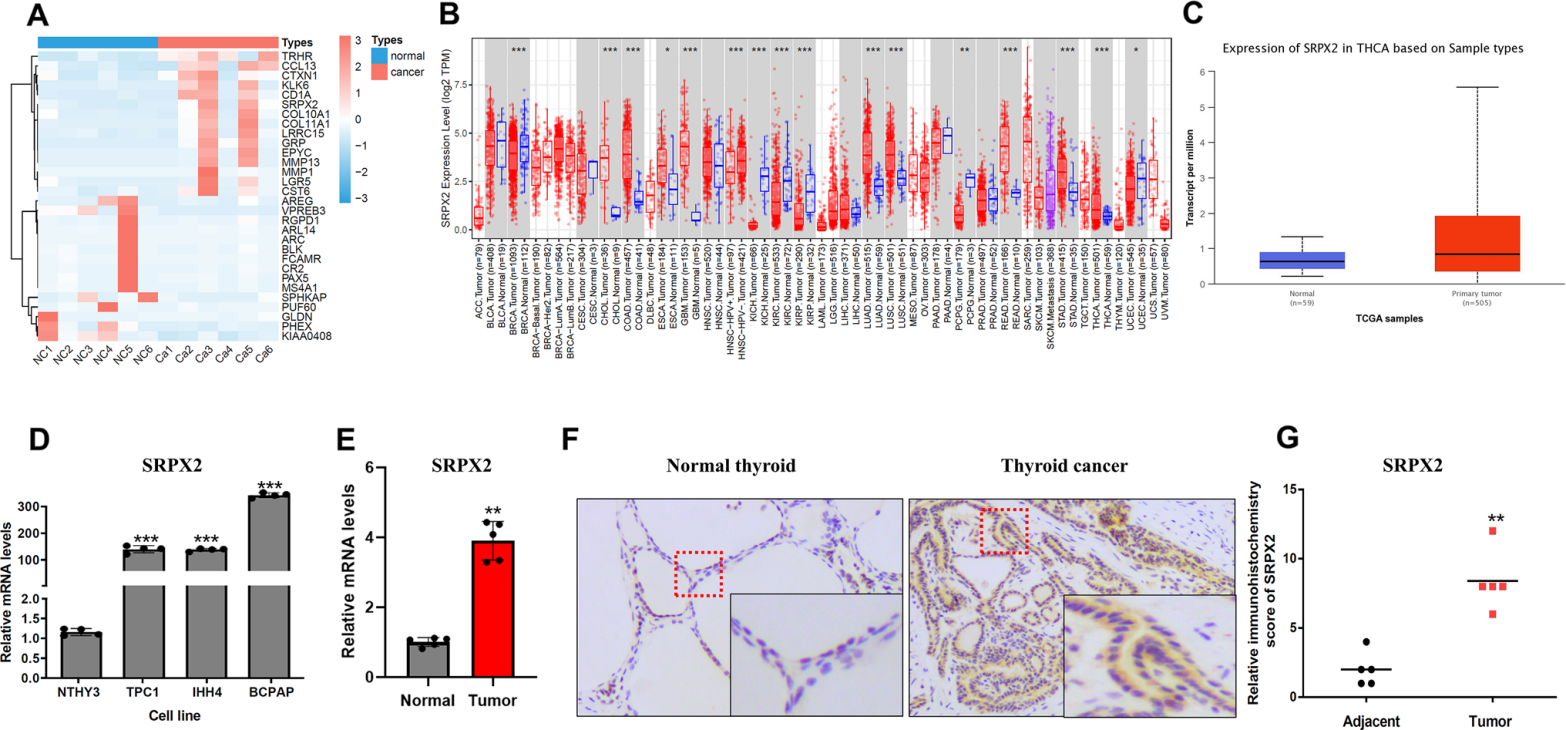
*SRPX2* shows high expression in thyroid cancer. **A** Six pairs of thyroid tumor samples and adjacent paracancerous tissues were sequenced. **B** The levels of *SRPX2* in different human tumor types were compared by Time2.0 database. **C** The significant increase in *SRPX2* expression in thyroid cancer was further validated by using the UALCAN cancer database. **D** RNA analysis of *SRPX2* expression in three human thyroid cancer cell lines (TPC1, IHH4, BCPAP) and a normal human thyroid cell line (NTHY3) (*P* = 0.0009). **E** Total RNA extracted from thyroid tumor specimens and adjacent paracancerous tissues was extracted for RT-qPCR analysis (*P* = 0.0079). **F** IHC image of *SRPX2* expression in thyroid cancer and paracancerous tissue. **G** The IHC score of *SRPX2* was measured in thyroid cancer and paracancerous tissues (*P* = 0.0011). All data were obtained from three independent experiments. *P < 0.05, **P < 0.01, ***P < 0.001

Six pairs of tumor tissues and adjacent non-tumor specimens were sequenced (Fig. 1A), and high-expression genes were selected for further study. We assessed the *SRPX2* levels based on RNA sequencing data from Time2.0 (http://timer.comp-genomics.org). In cancer tissues, *SRPX2* expression was higher than in normal tissues, especially in CHOL (cholangiocarcinoma), COAD (colon adenocarcinoma), ESCA (esophageal carcinoma), LUAD (lung adenocarcinoma), LUAC (lung adenocarcinoma), GBM (glioblastoma multiforme), READ (rectum adenocarcinoma), STAD (stomach adenocarcinoma), and THCA (thyroid carcinoma) (Fig. 1B). To verify *SRPX2* expression levels in thyroid cancer, we first analyzed the data from UALCAN (http://ualcan.path.uab.edu/index.html), which included 505 samples of thyroid cancer tissues and 59 thyroid normal tissues. *SRPX2* mRNA expression levels were higher in thyroid cancer tissues than in normal tissues according to UALCAN results (Fig. 1C, Supplementary Fig. 1). In addition, in the presence of the most common BRAF mutation in thyroid cancer, the expression of *SRPX2* was significantly higher than that in WT group (Supplementary Fig. 2). We further analyzed *SRPX2* mRNA levels in three human PTC cell lines (TPC1, IHH4, and BCPAP) (Fig. 1D). As shown in Fig. 1D, *SRPX2* mRNA levels were significantly increased compared with NTHY3. Besides, we extracted the total RNA from PTC tissues and paracancerous tissues of the six-paired samples, and *SRPX2* mRNA levels have shown to have a significant difference (Fig. 1E). To further confirm whether *SRPX2* expression is indeed upregulated in PTC, we performed immunohistochemical (IHC) analysis of *SRPX2* expression in cancer and paracancerous tissues. IHC also showed that *SRPX2* was highly expressed in PTC tissue compared with normal paracancerous tissue (Fig. 1F, G). Database analysis together with PCR and IHC results revealed that *SRPX2* expression is upregulated in PTC.

### High expression of *SRPX2* predicts a poor survival prognosis

**Fig. 2:**
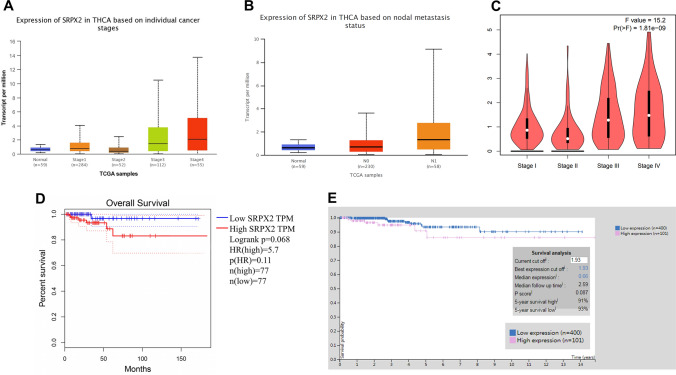
The expression level of *SRPX2* is related to the prognosis of thyroid cancer. **A**–**C** Boxplot graphs show the relative expression of *SRPX2* in all stages (**A**, **C**) and all lymph node metastasis grades **B** of thyroid cancer and normal tissues in the UALCAN (**A**, **B**) and GEPIA (**C**) database. **D**–**E** Kaplan–Meier survival curve shows that the overall survival rate of the patients with low *SRPX2* expression is better than that of the patients with high *SRPX2* expression. The GEPIA database suggests that the 5-year survival rates of high (*n* = 77) and low (*n* = 77) *SRPX2* expression levels were 85 and 98%, respectively (Logrank *p* = 0.068, HR (high) = 5.7, *p* (HR) = 0.11). The PROTEINATLAS database suggests that the 5-year survival rates of high (*n* = 101) and low (*n* = 400) *SRPX2* expression levels were 91 and 93%, respectively (*p* value = 0.087, median follow-up time: 2.59 months) (D: GEPIA database, E: PROTEINATLAS database)

To further evaluate the correlation between the expression level of *SRPX2* and the clinical significance in patients with thyroid cancer, we found higher *SRPX2* expression in all cancer stages and lymph node metastasis grades of thyroid cancer tissues than in normal tissues by using the UALCAN and GEPIA databases (http://gepia.cancer-pku.cn/index.html) (Fig. 2A-C). The GEPIA database was used to investigate the correlation between *SRPX2* expression and thyroid cancer prognosis. As a result, *SRPX2* expression significantly affected the prognosis of thyroid cancer patients (Fig. 2D). The Human Protein Atlas (https://www.proteinatlas.org) also revealed that patients with thyroid cancer who exhibited higher *SRPX2* expression (*n* = 400) have shorter survival time than those with lower expression patients (*n* = 101) (Fig. 2E). These results reveal that *SRPX2* is an important prognostic factor and has important clinical value in thyroid cancer.

### *SRPX2* knockdown inhibits the proliferation of PTC cells

**Fig. 3:**
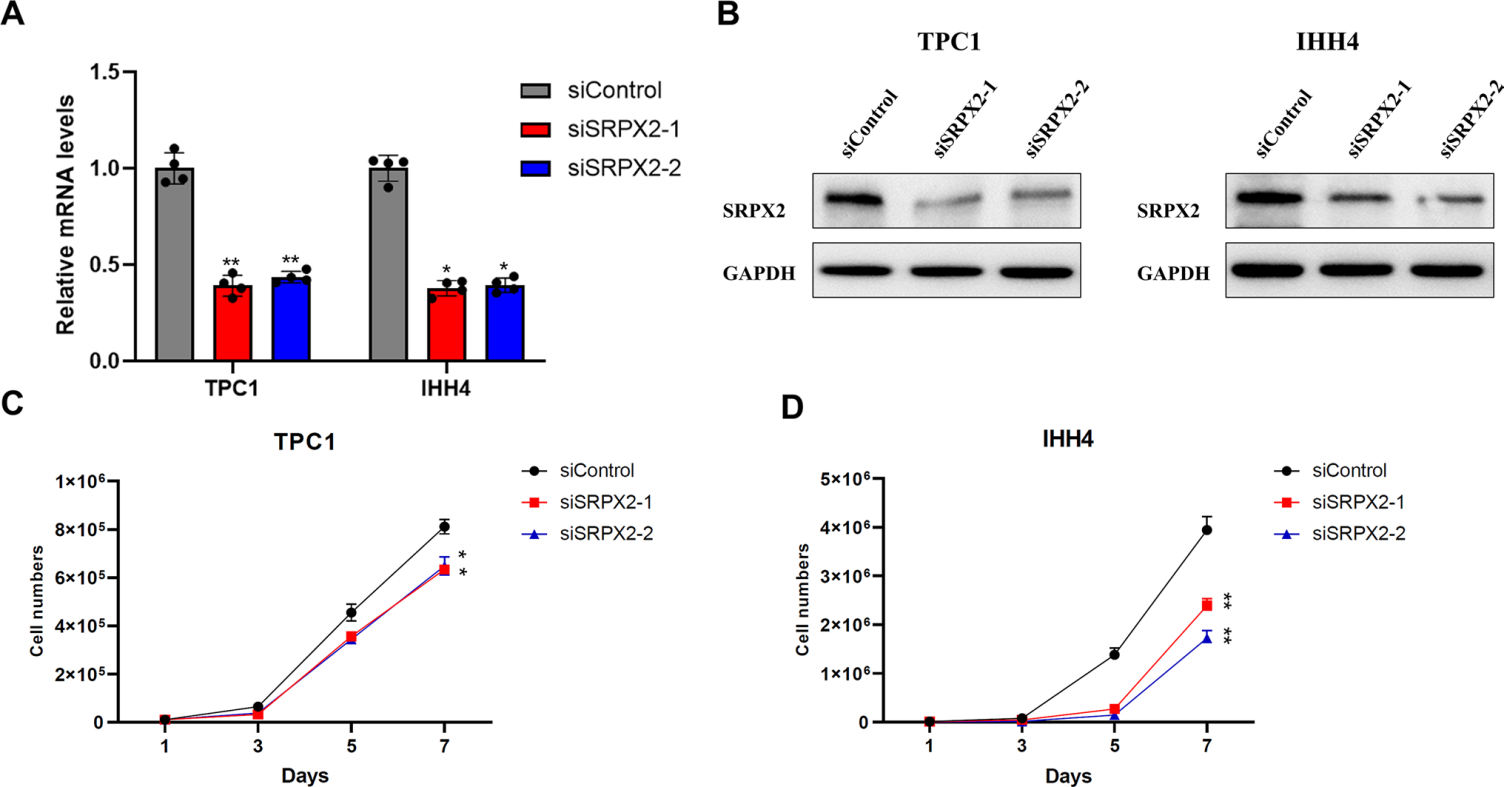
*SRPX2* knockdown reduces the proliferation ability of PTC cells. **A** RT-qPCR was used to verify the mRNA level of *SRPX2* in TPC1 (*P* = 0.0024) and IHH4 (*P* = 0.0107) cell lines after transfection for 24 h. **B** WB analysis showed that *SRPX2* was effectively knocked down by using siRNAs in TPC1 and IHH4 cells 72 h after cell transfection. **C**–**D** The growth curves of TPC1 (*P* = 0.0500) and IHH4 (*P* = 0.0036) cells. Cells were transfected with the siRNAs and then inoculated in plates at low density. The number of cells was calculated every other day. All data were obtained from three independent experiments. *P < 0.05, **P < 0.01, ***P < 0.001

To explore the cellular function of *SRPX2* in PTC, we designed two different siRNAs for *SRPX2* to carry out our experiments. RT-qPCR was used to detect the *SRPX2* mRNA level 24 h after transfection of TPC1 and IHH4 cells. The expression level of *SRPX2* has decreased (Fig. 3A). WB (Western blot) experiments also showed that *SRPX2* was effectively knocked down by two different siRNAs in the TPC1 and IHH4 cell lines (Fig. 3B). By using si*SRPX2*-1 and si*SRPX2*-2 as interference, we successfully generated PTC cell lines with low *SRPX2* expression. Next, we assessed the growth and proliferation of PTC cells in the si*SRPX2*-1 and si*SRPX2*-2 groups. We found that *SRPX2* inhibition resulted in growth inhibition of TPC1 and IHH4 cells (Fig. 3C, D).

### *SRPX2* inhibits PTC cell migration

**Fig. 4:**
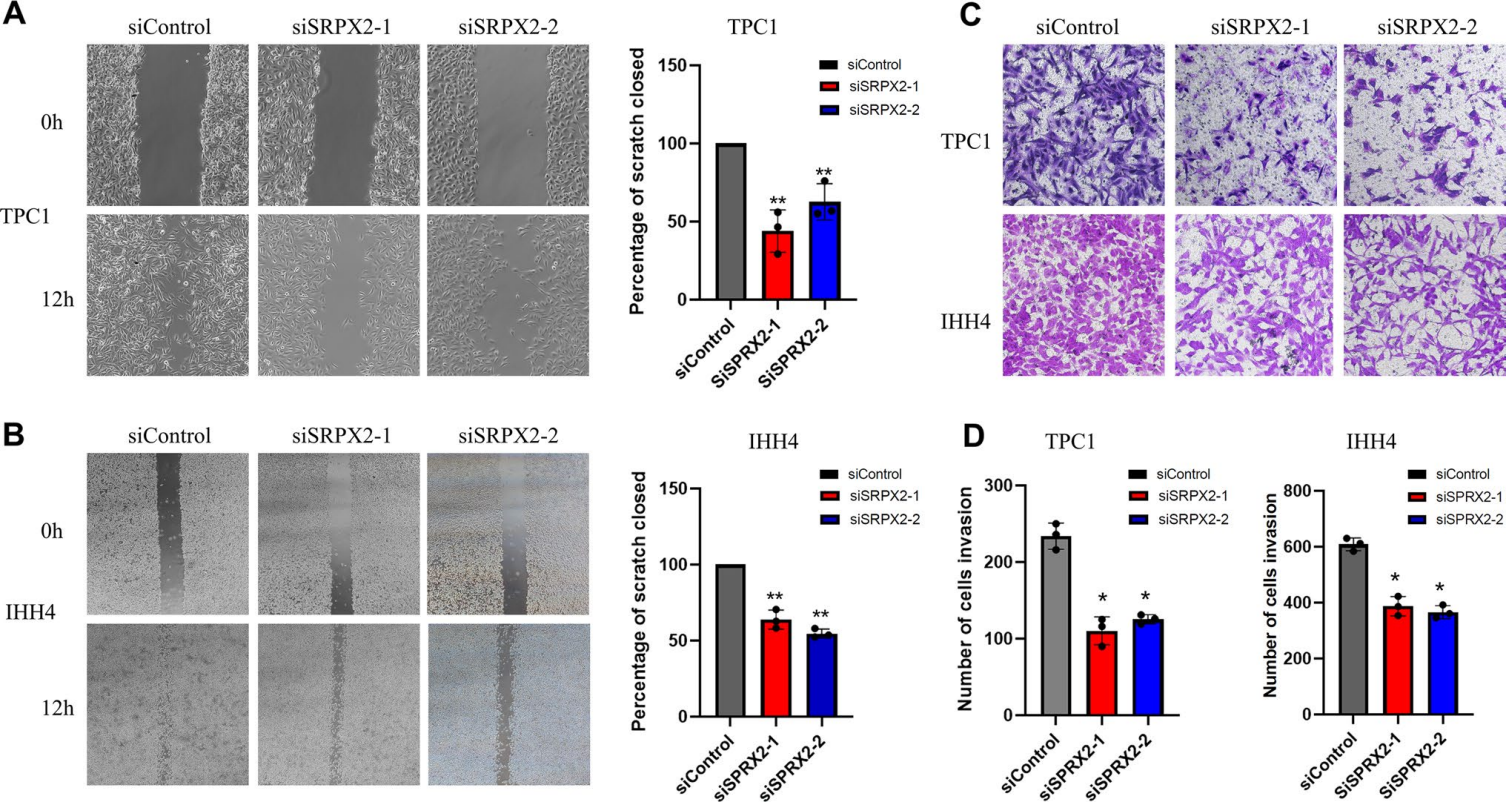
*SRPX2* knockdown reduces the migration ability of PTC cells. **A**–**B** The results of the wound healing experiment showed that cell migration ability was decreased within 12 h after *SRPX2* was knocked down in TPC1 (*P* = 0.0071) and IHH4 (*P* = 0.0036) cell lines. **C**–**D** The negative control group and *SRPX2* knockdown group were examined for TPC1 (*P* = 0.0107) and IHH4 (*P* = 0.0500) cell migration by Transwell. Transwell chamber was collected 24 h after laying. All data were obtained from three independent experiments. *P < 0.05, **P < 0.01, ***P < 0.001

To identify the function of *SRPX2* in the migration of PTC cells, we used siRNAs to knockdown *SRPX2* in TPC1 and IHH4 cells. After that, scratch assay showed that the downregulation of *SRPX2* inhibited the migration of TPC1 and IHH4 cells compared with control transfection (Fig. 4A, B). In addition, the Transwell assay also showed that silenced expression of *SRPX2* reduced the migration ability of TPC1 and IHH4 (Fig. 4C, D). Collectively, these results suggest that *SRPX2* promotes PTC tumor migration.

### *SRPX2*-related downstream molecules and signaling pathways

**Fig. 5:**
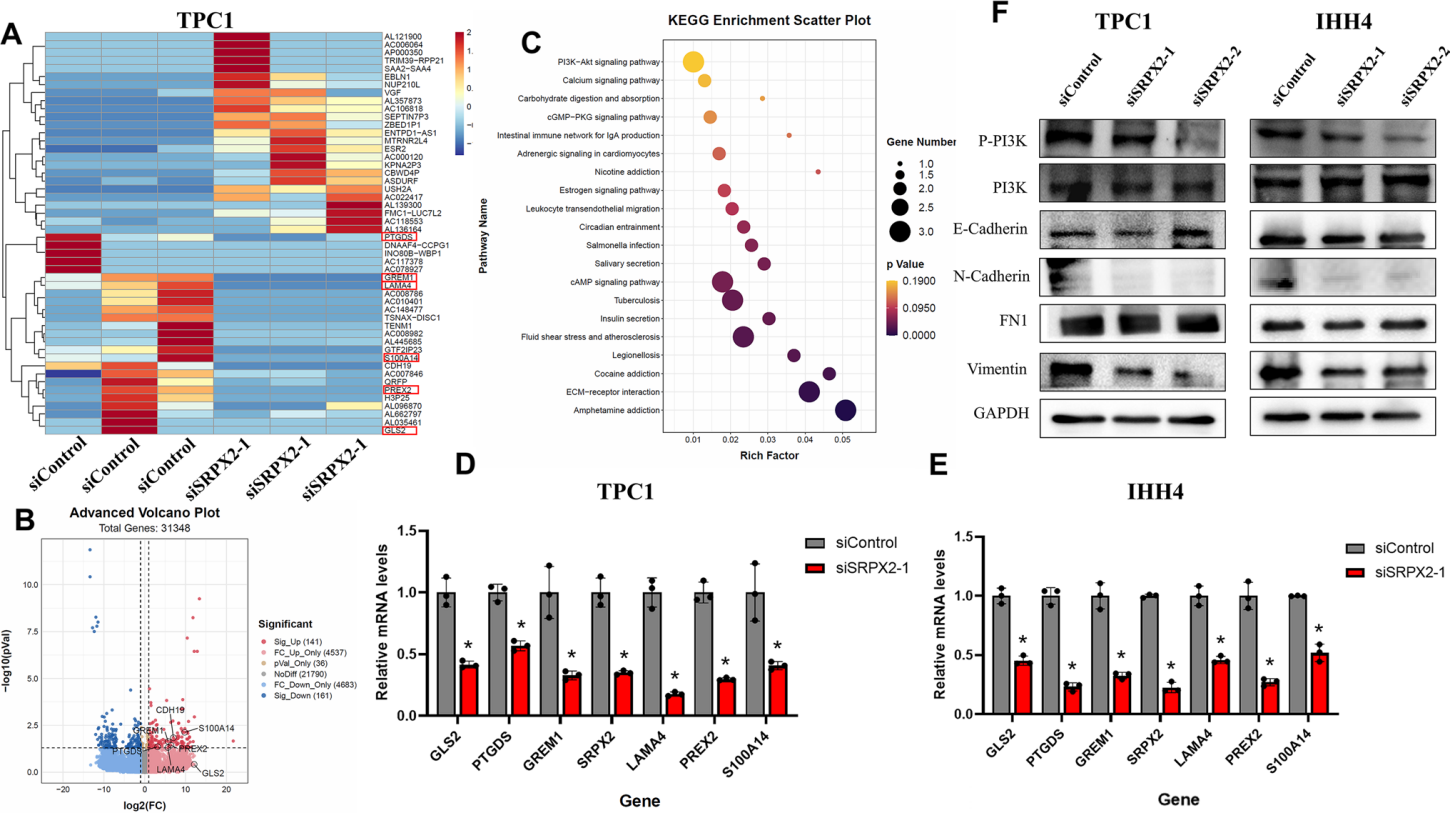
Pathways and downstream molecules involved in *SRPX2*. **A** RNA-seq Heat map of the gene expression profile form siControl and si*SRPX2*-1 treated TPC1 cells. The differentially expressed genes shown in the heat map were marked with red line. **B** The volcanic map shows all the genes associated with *SRPX2* in thyroid cancer. **C** KEGG analysis based on gene sequencing data was used to predict the potential function of *SRPX2* and the signal pathways involved. **D**–**E** Comparison of mRNA levels of downstream genes after knocking down *SRPX2* by RT-qPCR 24 h after transfection (*P* = 0.0156). **F** The P-PI3K, PI3K, and EMT-related protein (N-cadherin, E-cadherin, FN1, and vimentin) changes after *SRPX2* knockdown were verified by WB 72 h after cell transfection. *P < 0.05, **P < 0.01, ***P < 0.001

**Fig. 6:**
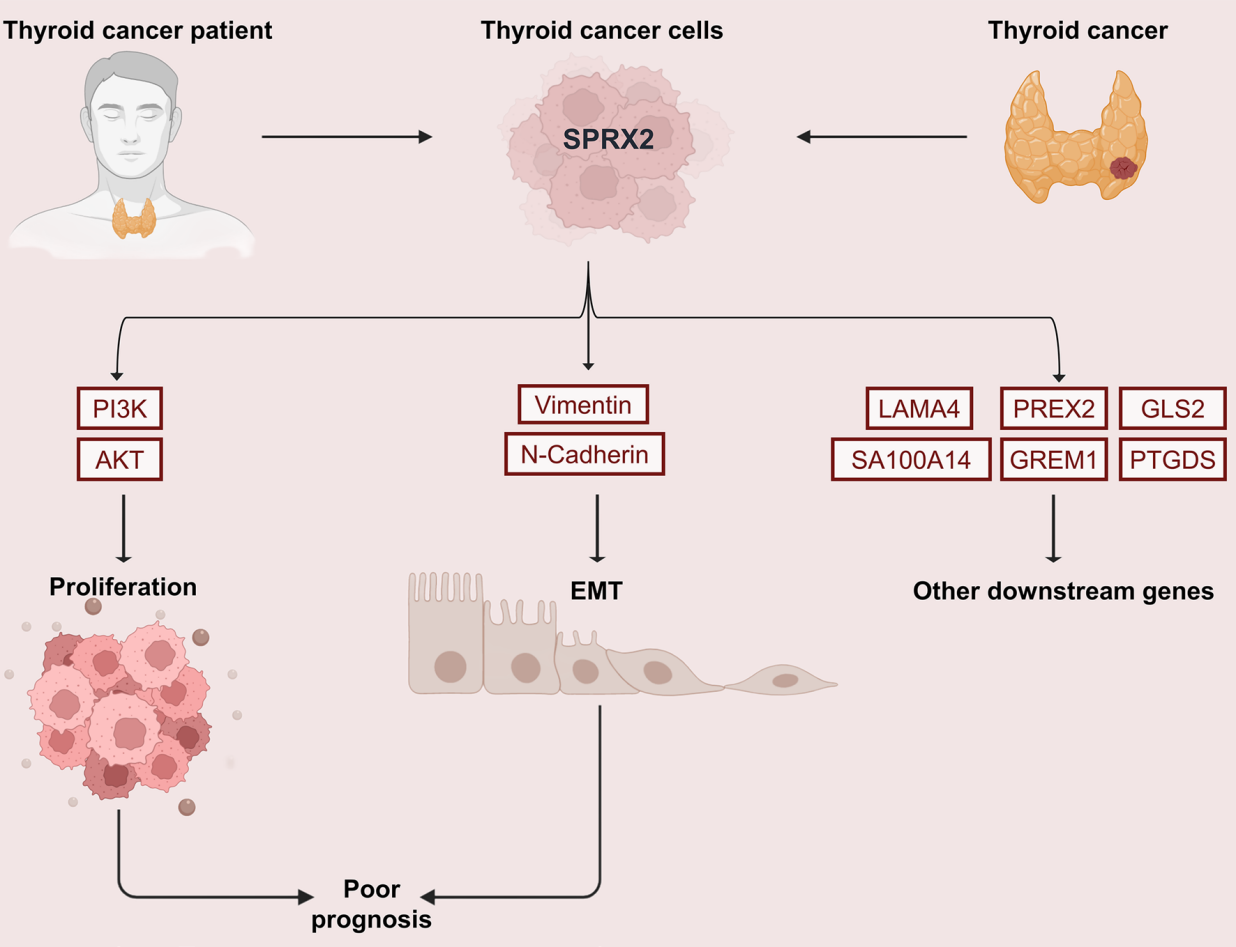
Molecular schematic diagram of *SRPX2* regulation

To identify potential downstream genes of *SRPX2*, we screened target genes using sequenced mRNAs. By comparing the gene expression changes between the control and *SRPX2* knockout groups, heatmap clustering analysis revealed that the expression of some genes decreased after the knockdown of *SRPX2*. Through literature review, we identified several *SRPX2* downstream genes with potential clinical value, including *PTGDS, GREM1, LAMA4, S100A14, PREX2,* and *GLS2* (Fig. 5A). Based on gene sequencing, we generated a volcanic map of all *SRPX2*-associated genes and found a positive correlation between the levels of *PTGDS, GREM1, LAMA4, S100A14, PREX2, GLS2,* and *SRPX2* (Fig. 5B). We then performed KEGG analysis of the *SRPX2* gene and identified the top 20 KEGG-enriched terms (Fig. 5C). The KEGG analysis revealed that *SRPX2* was highly correlated with the *PI3K/AKT* signaling pathway, calcium signaling pathway, amphetamine signaling pathway, and ECM receptor interaction. Taken together, these findings suggest that *SRPX2* mediates a range of malignant biological functions in PTC cell lines by activating the *PI3K/AKT* pathway and downstream target genes. In order to verify our hypothesis that *SRPX2* is directly proportional to the expression of downstream target genes, we performed *SRPX2* knockdown in the TPC1 and IHH4 cell line and verified it by RT-qPCR. As expected, the mRNA levels of *PTGDS, GREM1, LAMA4, S100A14, PREX2,* and *GLS2* decreased when *SRPX2* was effectively knocked down (Fig. 5D, E). Previous studies have suggested that *SRPX2* is involved in *PI3K/AKT* pathway, suggesting that *SRPX2* may be involved in tumor metastasis. The results of the Transwell experiment also showed that after the *SRPX2* gene interfered, the metastatic ability of PTC cells was decreased. This suggests that the *SRPX2* protein may be one of the key regulatory factors for PTC cell metastasis. We next examined the protein level of vimentin, N-cadherin, E-cadherin, and FN1, which are typical epithelial–mesenchymal transition (EMT) regulating proteins. We found that *SRPX2* knockdown resulted in decreased levels of *N-cadherin* and *vimentin* in the TPC1 and IHH4 cell lines, whereas the protein levels of *E-cadherin* and *FN1* did not change significantly (Fig. 5F). At the same time, WB results showed that *P-PI3K* levels were decreased after *SRPX2* knockdown, whereas *PI3K* protein levels were not significantly changed (Fig. 5F). These results suggest that *SRPX2* regulates PTC via the *PI3K/AKT* pathway and promotes EMT via *N-cadherin* and *vimentin*. We also identified several *SRPX2* downstream genes, including *PTGDS, GREM1, LAMA4, S100A14, PREX2,* and *GLS2*, which require further experiments to identify the detailed mechanisms (Fig. 6).

## Discussion

In recent years, the incidence rate of thyroid cancer has increased, and the new cases of thyroid carcinoma with a larger diameter and non-early cancer have shown an upward trend [25, 26]. In addition to the continuous increase in the prevalence rate, it is also a hot research topic that the prognosis of PTC is difficult to judge and there is no recognized optimal treatment [27]. Presently, benign and malignant thyroid nodules are mainly differentiated using traditional ultrasound and needle aspiration biopsy [28, 29]. However, due to the varying experience of the operators and the location of sampling, there is a misdiagnosis rate in clinical practice. Currently, there are no clear markers for thyroid cancer. Therefore, identifying molecular markers of thyroid cancer is of great significance for the early diagnosis, treatment, and prevention of postoperative recurrence of thyroid cancer.

Our study identified a meaningful gene that could affect PTC prognosis through extensive bioinformatics analysis. We found that *SRPX2* was overexpressed in PTC and positively correlated with higher histological grade, metastatic lymph node grade, and worse prognosis. Previous studies have found that *SRPX2* takes part in human embryonic stem cell differentiation, cognitive impairment, and epileptic activity [19, 30, 31]. Most published data show that *SRPX2* has a carcinogenic effect and is abnormally expressed in various tumors [15, 32, 33]. Our study also showed that the mRNA level of *SRPX2* in PTC tissues was significantly higher than paracancerous tissues, which was confirmed by cell experiments and IHC. All these results suggest that *SRPX2* is a prognostic biomarker of PTC.

Yu et al. found that *SRPX2* targets the *FAK/AKT* pathway in hepatocellular carcinoma (HCC) to increase the mobility of HCC cells [34], and *SRPX2* promotes EMT in small cell lung cancer [14]. In addition, previous studies have shown that *SRPX2* partially realizes its function through the *FAK*-dependent pathway, and *SRPX2* targets *FAK* to exert malignant biological effects in thyroid cancer [35]. However, studies on *SRPX2* in thyroid cancer are rare and the mechanism of action of *SRPX2* in thyroid cancer remains unclear. Our article is the first showing that *SRPX2* enhances the cell proliferation and migration ability of PTC by regulating the *PI3K/AKT* signaling pathway, *N-cadherin, *and* vimentin* and additionally explores more potential regulating genes. Through KEGG analysis, we confirmed that the expression of *SRPX2* was closely related to the *PI3K/AKT* signaling pathway, and WB verified that the level of *P-PI3K* decreased after knocking down *SRPX2.* Furthermore, the results of thermal cluster analysis and the volcanic map of *SRPX2* showed that the expression of *PTGDS, GREM1, LAMA4, S100A14, PREX2,* and *GLS2* in thyroid carcinoma was positively correlated with *SRPX2*. In addition, the RT-qPCR results showed that their mRNA levels decreased to different degrees after *SRPX2* knockdown. Other studies have also shown that *PTGDS, GREM1, LAMA4*, *S100A14, PREX2,* and *GLS2* have potential value in promoting cancer progression and predicting cancer prognosis [36–41]. This is the first research to reveal a direct correction between *SRPX2* and these oncogenes, which needs further study.

Our study had some limitations. The number of patients included in this study was limited, and it is difficult to explain the differences in age, race, and geographical distribution. Therefore, additional in vivo and in vitro experiments are required for verification.

In conclusion, our study shows that *SRPX2* is highly expressed in PTC and is involved in tumor progression. An increase in *SRPX2* expression is associated with a decrease in overall survival in patients with PTC and may serve as an independent prognostic factor. In addition, *SRPX2* promotes the malignancy of PTC through the *PI3K/AKT, N-cadherin*, and *vimentin* pathways. We also identified potential downstream regulatory genes, including *PTGDS, GREM1, LAMA4, S100A14, PREX2,* and *GLS2*. Therefore, our study offers new insights into the role and mechanism of *SRPX2* in thyroid cancer and its potential as a biomarker for PTC prognosis.

### Supplementary Information

Below is the link to the electronic supplementary material.

Supplementary file1 (TIF 565 KB)

Supplementary file2 (TIF 1741 KB)

Supplementary file3 (DOC 36 KB)

## Data Availability

RNA data are available at the Gene Expression Omnibus datasets (accession number: GSE233267). The data that support the findings of this study are available from the corresponding author upon reasonable request.
