# Supplementary figures and images for: SRPX2 promotes cancer cell proliferation and migration of papillary thyroid cancer
Source: Clin Exp Med. 2023 Jun 12;23(8):4825–34. doi: 10.1007/s10238-023-01113-1 (PMC10725347; doi:10.1007/s10238-023-01113-1)

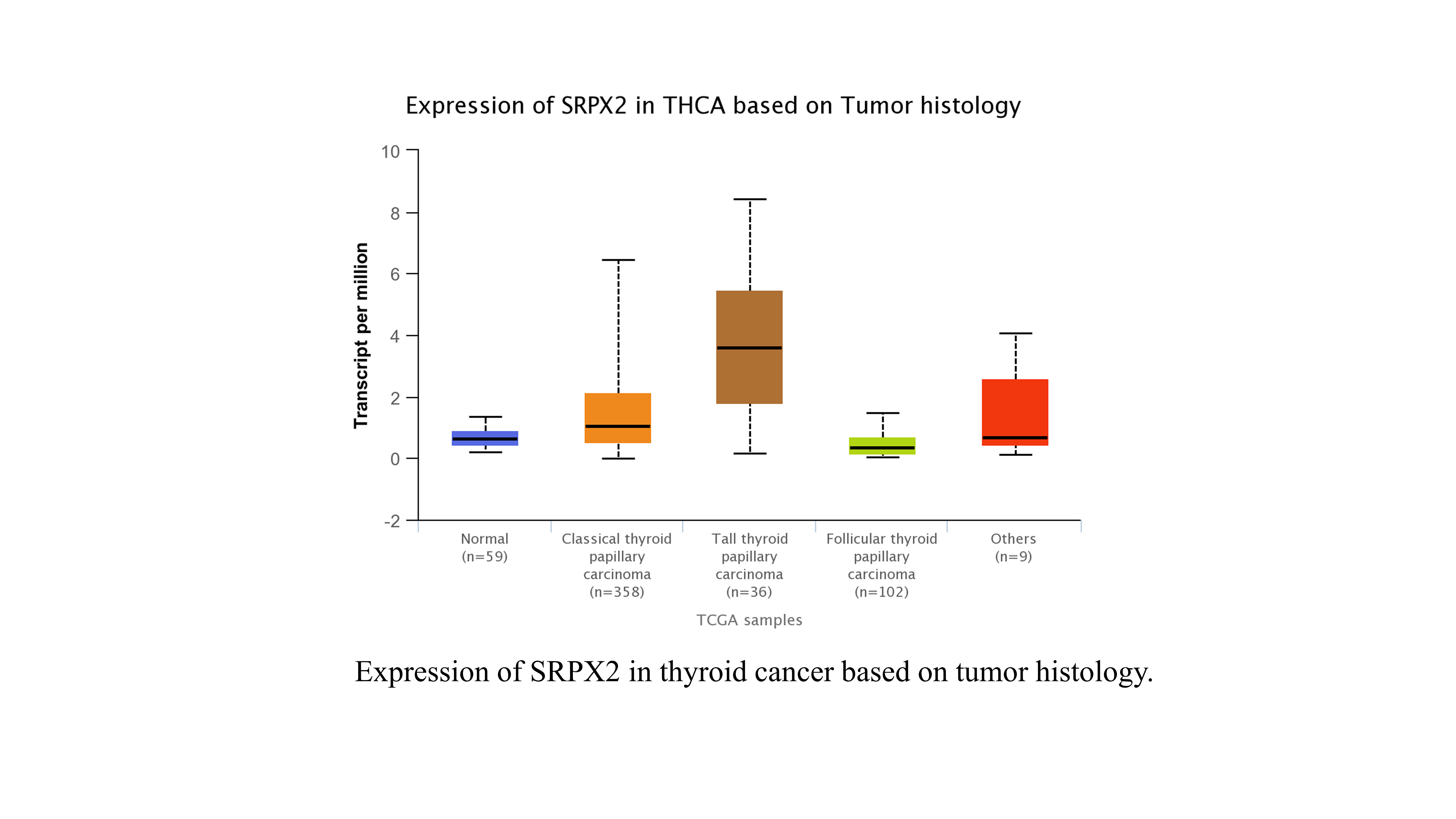

Supplement: Supplementary file 1 — Supplementary file1 (TIF 565 KB) [file 10238_2023_1113_MOESM1_ESM.tif]

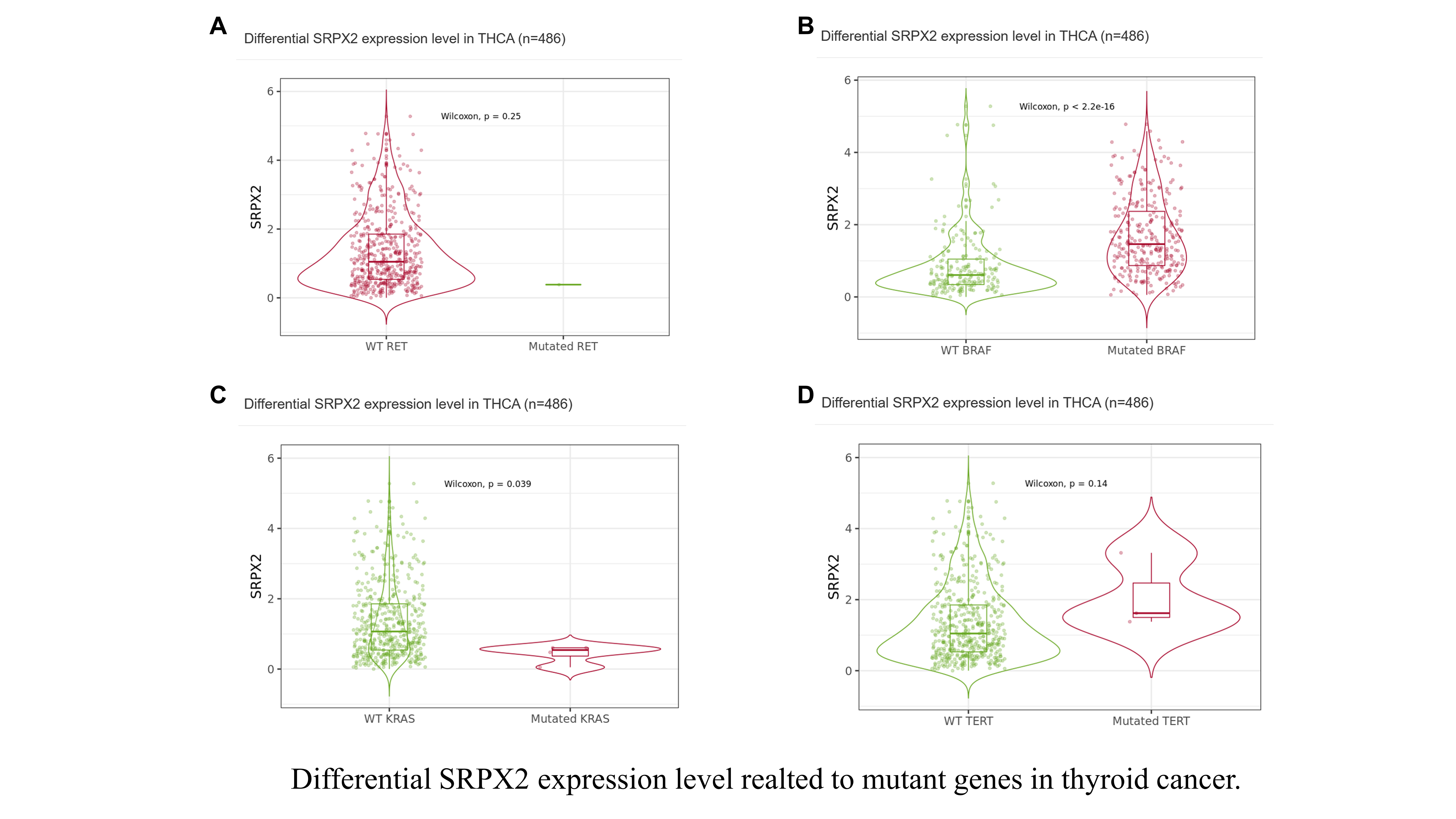

Supplement: Supplementary file 2 — Supplementary file2 (TIF 1741 KB) [file 10238_2023_1113_MOESM2_ESM.tif]
