# Supplementary material for: SRPX2 promotes cancer cell proliferation and migration of papillary thyroid cancer
Source: Clin Exp Med. 2023 Jun 12;23(8):4825–34. doi: 10.1007/s10238-023-01113-1 (PMC10725347; doi:10.1007/s10238-023-01113-1)
Supplement: Supplementary file 3 — Supplementary file3 (DOC 36 KB) [file 10238_2023_1113_MOESM3_ESM.doc]

Supplementary Table 1: Basic information of the six thyroid cancer patients.

| Sample  ID | Sex | Age | Tumor size | Cancer histological type | T stage | N stage | M stage | LNR | ETE | Multifocality | Vascular/nerve invasion | TNM stage |
| --- | --- | --- | --- | --- | --- | --- | --- | --- | --- | --- | --- | --- |
| 1 | Female | 30 y | 0.8 cm | Papillary thyroid carcinoma | T1a | N1b | M0 | 4/51 | no | no | no | I |
| 2 | Female | 38 y | 1.8 cm | Papillary thyroid carcinoma | T3b | N1b | M0 | 9/22 | yes | yes | no | I |
| 3 | Female | 46 y | 1.3 cm | Papillary thyroid carcinoma | T1b | N1b | M0 | 4/23 | yes | no | no | I |
| 4 | Female | 23 y | 1.2 cm | Papillary thyroid carcinoma | T1b | N1a | M0 | 1/4 | no | no | no | I |
| 5 | Female | 58 y | 1.3 cm | Papillary thyroid carcinoma | T1b | N0 | M0 | 0/3 | no | no | no | I |
| 6 | Female | 34 y | 2.5 cm | Papillary thyroid carcinoma | T3b | N1a | M0 | 1/6 | yes | no | yes | I |

Y: year; LNR: lymph node ratio; ETE: extrathyroidal extension;
